# Psychiatric and Cognitive Functioning After Metabolic and Bariatric Surgery: A Systematic Review and Meta‐Analysis

**DOI:** 10.1111/obr.13968

**Published:** 2025-06-22

**Authors:** Emma A. van Reekum, Michael Darcy, Jaslyn Drage, Joshua Xu, Kimberly Ng, Benjamin Forestell, Nancy Santesso, Raed A. Joundi, Jorge Wong, Aristithes Doumouras, Valerie H. Taylor, Salim Yusuf, Ryan Van Lieshout

**Affiliations:** ^1^ Department of Psychiatry and Behavioural Neurosciences, Faculty of Health Sciences McMaster University Hamilton Ontario Canada; ^2^ Department of Health Research Methods, Evidence, and Impact, Faculty of Health Sciences McMaster University Hamilton Ontario Canada; ^3^ Population Health Research Institute McMaster University and Hamilton Health Sciences Hamilton Ontario Canada; ^4^ Department of Psychiatry University of Toronto Toronto Ontario Canada; ^5^ Department of Surgery University of Calgary Calgary Alberta Canada; ^6^ Emergency Medicine, Faculty of Health Sciences McMaster University Hamilton Ontario Canada; ^7^ Department of Surgery, Faculty of Health Sciences McMaster University Hamilton Ontario Canada; ^8^ Department of Psychiatry University of Calgary Calgary Alberta Canada

**Keywords:** cognitive function, mental health, metabolic and bariatric surgery, obesity

## Abstract

**Objective:**

To systematically review and meta‐analyze psychiatric and cognitive outcomes following metabolic and bariatric surgery (MBS). [Correction added on 1 July 2025, after first online publication: The objective statement has been updated for clarity.]

**Methods:**

Six databases were searched. Randomized controlled trials (RCTs) and nonrandomized studies (NRS) of people with obesity comparing MBS with any nonsurgical intervention or control condition were included. Main outcomes included symptoms of depression, anxiety, and non‐normative eating, substance use disorder diagnosis, suicide death, and cognitive performance in attention, memory, and executive function. Evidence certainty was assessed with GRADE. Heterogeneity was explored with subgroup analyses of ≤ 2 years vs. > 2 years post‐intervention.

**Results:**

There were 79 studies (75 NRS and 4 RCTs) found, including 732,149 people with obesity who underwent MBS, and 7,670,770 who did not. Among RCTs, MBS may improve depressive symptoms (standardized mean difference [SMD] = −0.40, 95% CI −1.04, 0.24; very low certainty). Among NRS, there was low to very low certainty that MBS may improve depressive (SMD = 0.56, 95% CI −0.87, −0.26), anxiety (SMD = −0.60, 95% CI −1.00, −0.19), and non‐normative eating symptoms (SMD = −0.75, 95% CI −0.97, −0.53) and cognitive performance in attention (SMD = −0.72, 95% CI −1.61, 0.17), but not executive function or memory. MBS may slightly increase suicide deaths (1/1000 more people, 95% CI 0 fewer to 3 more; very low certainty) and substance use disorders (4/100 more, 95% CI from 1 to 9 more; low certainty) > 2‐years post‐surgery.

**Conclusions:**

Compared to nonsurgical conditions, MBS may improve depression, anxiety, non‐normative eating, and attention, but slightly increase suicides and substance use disorders. There was low to very low certainty in most outcomes, therefore additionalhigh‐quality studies are needed to strengthen the evidence base.

## 1 Introduction

Obesity is a chronic metabolic disease involving excess adipose tissue that has the potential to impair health [1]. It currently affects 14% of the global population and is projected to rise to 24% by 2035 [2, 3]. Because of its known health consequences, obesity is expected to be one of the most serious global challenges of the 21st century [4].

There is substantial evidence that obesity negatively affects the brain, manifesting as reduced psychiatric and cognitive functioning and greater prevalence of diagnosed psychiatric disorders. While imperfectly understood, there are many proposed mechanisms linking obesity to changes in the brain, such as endothelial dysfunction, elevated inflammation and cortisol, vascular risk factors, weight‐based discrimination, and pain conditions [5, 6, 7, 8]. Indeed, longitudinal studies suggest that early‐ and mid‐life obesity independently increases the risk for incident depressive disorders (odds ratio [OR] = 1.57, 95% CI 1.23–2.01), anxiety disorders (OR = 1.86, 95% CI 1.03–3.37), and dementia (risk ratio [RR] = 1.41, 95% CI 1.20–1.66) [5, 9, 10]. Sabia and colleagues also found that obesity was associated with a more rapid decline in general cognitive functioning, memory, and executive function over time [11].

Many studies have found a high prevalence of psychiatric disorders in people with obesity [12, 13, 14]. A meta‐analysis pooling studies that diagnosed psychiatric disorders using different methods found that depressive disorders, eating disorders, and anxiety disorders were most common, affecting 19%, 17%, and 12% of individuals seeking obesity treatment, respectively [12]. However, prevalence estimates vary between studies and may be influenced by the study population and diagnostic approach used. For example, a review of studies that employed structured diagnostic interviews found that anxiety disorders may be most common, affecting between 12‐24% of people [13]. Using this diagnostic approach, Duarte‐Guerra and colleagues found that 58% of people seeking obesity treatment had a current psychiatric disorder, and 81% had experienced a disorder in their lifetime, though other studies have found lower estimates [13, 14].

Psychiatric disorders and their mental health and cognitive symptoms cause significant burden to affected individuals and communities, increasing disability and reducing quality and quantity of life [15, 16, 17]. Interventions are needed that can reduce the impact of these problems in the growing number of people with obesity.

Metabolic and bariatric surgery (MBS) is the most effective and durable obesity treatment, with large prospective studies finding ~30% total body weight loss that is generally sustained over time [18, 19, 20, 21]. While new obesity medications like glucagon‐like peptide‐1 receptor agonists and glucose‐dependent insulinotropic polypeptide agonists may lead to up to 21‐26% total body weight loss, almost 85% of people may experience weight recurrence when medications are stopped, side effects can be burdensome or intolerable, and their current costs can be prohibitive [21, 22]. MBS remains a treatment of choice for severe and/or refractory obesity and for people who prefer not to take medications indefinitely or who require rapid improvement in obesity or its sequelae [20]. Of the existing obesity treatments, MBS has the strongest evidence for improving health consequences of obesity [21]. Globally, 600,000 people receive this intervention yearly, and demand is increasing [23, 24].

Existing evidence supports the positive psychiatric and cognitive effects of MBS, though there are important discrepancies and limitations. Most robust are findings of improved depression after MBS from several recent systematic reviews with and without meta‐analysis. In the largest meta‐analysis conducted to date, Fu and colleagues found that depressive symptoms improved and the prevalence of depression was reduced after surgery [25]. Results aligned with a separate meta‐analysis by Loh and colleagues and a narrative synthesis [26, 27]. However, other narrative syntheses had contrasting conclusions, in particular that improvements in depression persist 2 years after MBS or that symptoms may return after this time [28, 29].

Previous systematic reviews have also examined anxiety, non‐normative eating, and cognitive performance after MBS. A meta‐analysis found that anxiety symptoms improved, and the prevalence of anxiety decreased after surgery, whereas conflicting findings were found in narrative syntheses [26, 28, 29]. Three systematic reviews without meta‐analysis examined different forms of non‐normative eating and suggested that there are at least short‐term improvements in night eating, binge eating, emotional eating, and grazing after MBS, compared to before [28, 30, 31]. Li and colleagues reported meta‐analytic evidence that compared to no intervention, MBS improves cognitive performance in general memory, but not executive function or attention, and results were similar in 2 narrative syntheses [32, 33, 34].

In contrast, some recent reviews have reported psychiatric adverse events after MBS. In a meta‐analysis, alcohol use disorder prevalence up to 2 years after surgery was comparable to pre‐surgical levels, but increased after this time [35]. Two further narrative syntheses aligned with these results and suggested that other types of substance use may increase after surgery [36, 37]. Another meta‐analysis found elevated rates of suicide and self‐harm in people after MBS when compared to nonsurgical control groups and their own pre‐surgical rates [38]. While a narrative synthesis aligned with findings of increased suicide compared to general population estimates, a different meta‐analysis found comparatively less suicide in people who received MBS [39, 40].

There are several limitations of this work. Existing reviews have generally not followed a registered protocol, which can increase the risk of reporting bias [25, 26, 27, 29, 31, 32, 34, 35, 36, 37, 38, 39]. Relevant studies may also have been missed due to search restrictions by language, exclusion of gray literature, and the fact that some studies only searched 1 or 2 databases, excluded people with comorbidities, excluded older studies, orexcluded randomized controlled trials (RCTs) [25, 26, 27, 28, 29, 30, 31, 32, 34, 35, 36, 37, 38, 39]. Meta‐analyses were not always done. Subgroup analyses were usually not pre‐specified and heterogeneity either remained high and/or was not reported [25, 26, 35]. Within‐groups (before‐after) studies were most commonly examined, which could add bias [25, 26, 27, 28, 29, 30, 31, 32, 33, 35, 36, 37, 38, 41, 42]. For example, depressive symptoms may spontaneously improve (i.e., regression to the mean), cognitive tests may have a practice effect, and people may under‐report or temporarily suppress substance use before obesity treatment to meet eligibility criteria. When between‐groups studies were examined, some reviews pooled studies that reported on the same study population, and control groups had limitations (such as obesity registries or general populations) [32, 34, 39]. In general, reviews either did not examine the risk of bias of included studies or used approaches that are not aligned with best practices [25, 26, 27, 28, 29, 30, 31, 32, 33, 34, 35, 36, 37, 38, 39]. Reviews usually presented relative and not absolute effects, and rarely assessed evidence certainty, making interpretability and clinical decision‐making based on them difficult. Indeed, 2 umbrella reviews rated the quality of systematic reviews in this field to be mostly of low or critically low quality [25, 26, 27, 28, 29, 30, 31, 32, 33, 34, 35, 36, 37, 38, 43, 44].

Furthermore, an updated systematic review and meta‐analysis that contains a broad range of psychiatric and cognitive outcomes after MBS is needed. Present reviews on this topic usually assess 1 or 2 psychiatric disorders and/or their symptoms, requiring clinicians who care for people with obesity to be aware of a large body of literature. To the authors' knowledge, no meta‐analysis of RCTs has compared psychiatric and cognitive outcomes between surgical and nonsurgical groups, and at least two RCTs have been conducted [45, 46].

Thus, this systematic review and meta‐analysis synthesized the literature to determine what is known about the effect of MBS on psychiatric and cognitive functioning in people with obesity, compared to nonsurgical groups.

## 2 Methods

### 2.1 Search Strategy and Selection Criteria

Six databases were searched (Medline, Embase, EMCare, Web of Science, PsychINFO, and CENTRAL) from inception to August 4, 2023 (Methods S1 in the Supporting Information).

Studies were included if they examined people with obesity, compared MBS to any nonsurgical obesity intervention or other control condition (i.e., compared between‐groups), and assessed mental health, cognition, psychiatric disorders, or their treatment. Nonrandomized studies (NRS; excluding within‐groups studies) and RCTs were eligible but were separately analyzed. Obesity was defined according to individual study criteria. All MBS procedures were eligible except for revisional surgeries. Active (lifestyle intervention and medical weight management including medication) and/or passive conditions (surgical waitlists, obesity registries, and no formal intervention) could serve as controls. Psychiatric and cognitive outcomes were assessed using validated scales, structured interviews, and/or medical record data. Given definitional and diagnostic challenges surrounding eating disorders after MBS, levels of non‐normative eating symptoms and proportions of people with an eating disorder were examined, with the latter defined using any validated, dichotomous measure of eating pathology [47, 48]. No exclusions were made based on language. Non‐English studies were translated prior to data extraction. Data were sought from published manuscripts or abstracts. Individual participant‐level data were not requested.

Two investigators (EvR, MD, JD, JX, and KN) independently screened studies, and disagreements were resolved either by consensus or by involving a senior reviewer (EvR) if she was not involved in the disagreement. If she was involved, then RV resolved disagreements. Two investigators (EvR, MD, JD, JX, KN, and BF) independently extracted data, and disagreements were resolved by consensus at this stage.

### 2.2 Outcomes

Main outcomes were chosen for clinical relevance, including depressive symptoms, anxiety symptoms, non‐normative eating symptoms, diagnosis of a substance use disorder, suicide death, and cognitive performance on tests of attention, memory, and executive function. Other outcomes included the proportion of people with diagnosed depressive disorders, anxiety disorders, eating disorders, dementia, any psychiatric disorder, and proportions of people with suicidal ideation/self‐harm behavior, psychiatric treatment use, and psychiatric hospitalizations, as well as levels of substance use symptoms.

### 2.3 Data Analysis

Data extraction was done in Covidence using piloted extraction forms [49]. When studies reported more than 1 time point, the most distal data was extracted. When authors confirmed that identical data existed in 2 or more papers, the publication with the largest sample size and longest follow‐up was selected. An a priori created hierarchy based on clinical relevance to people with obesity guided data selection for analyses when studies reported more than 1 method of outcome assessment (Table S1).

Meta‐analyses were conducted when at least 2 study populations contained meta‐analyzable data. Unadjusted data were pooled because few NRS provided adjusted results, and those that did contained disparate covariates. Data analysis was conducted in Review Manager (RevMan) v5.4.1 [50]. Random‐effects models were used for all meta‐analyses; however, fixed effects models were used and reported if there were small study effects (i.e., when small studies biased the meta‐analysis). Standardized mean differences (SMDs), mean differences (MDs), or risk ratios (RRs) and their 95% confidence intervals (95% CI) were calculated. RRs were transformed to absolute risks using the control group event rate as the baseline risk. Number needed to treat (NNT) or harm (NNH) was also presented. Heterogeneity was evaluated statistically with the I^2^ statistic and explored with a pre‐specified subgroup analysis by year after surgery (≤ 2 years vs > 2 years), as weight and comorbidity recurrence may begin 2 years after surgery [51]. Only statistically significant subgroup effects are presented. A priori planned sensitivity analyses removing studies at critical risk of bias and studies with passive control groups (i.e., surgical wait‐lists, obesity registries, no formal intervention) were conducted since active interventions may substantially reduce weight and so are a more meaningful comparison to surgery [20]. A post hoc sensitivity analysis removing 1 study [52] that used a duodenal‐jejunal bypass liner (DJBL) intervention was done, as it reduces weight less than other MBS procedures but more than some nonsurgical options. Narrative syntheses were provided for studies with insufficient data or that were not meta‐analyzable, using combined *p*‐value and vote counting approaches [53].

### 2.4 Risk of Bias

Risk of bias was assessed with the Cochrane RoB2 for RCTs and the ROBINS‐I for NRS [54] (p2) [55]. To assess confounding bias in ROBINS‐I, the following potential confounders were pre‐specified based on past research: age, sex or gender, education, socioeconomic status or income, psychiatric comorbidity, medical comorbidity, and obesity severity. Studies were determined to be at serious risk of bias when they controlled for only some of these factors, and at critical risk of bias when none were considered.

### 2.5 Evidence Certainty

Evidence certainty was assessed according to the Grading of Recommendations Assessment, Development and Evaluation (GRADE) framework [56]. Appreciable benefit and harm thresholds were established to evaluate imprecision and interpret results. For continuous outcomes, this threshold was set at 0.2 SMD. For dichotomous outcomes, an absolute difference in 1/1000 people was considered meaningful for the suicide death outcome, and 2/100 people was meaningful for other outcomes. For the Alcohol Use Disorders Identification Test (AUDIT), a change of ≥ 3 points was considered clinically important [57]. Publication bias was explored using funnel plots if there were ≥ 10 studies.

### 2.6 Registration and Reporting

The review was preregistered on PROSPERO (CRD 42022358360). The Preferred Reporting Items for Systematic Reviews and Meta‐analyses (PRISMA) guideline was followed [58].

## 3 Results

**FIGURE 1:**
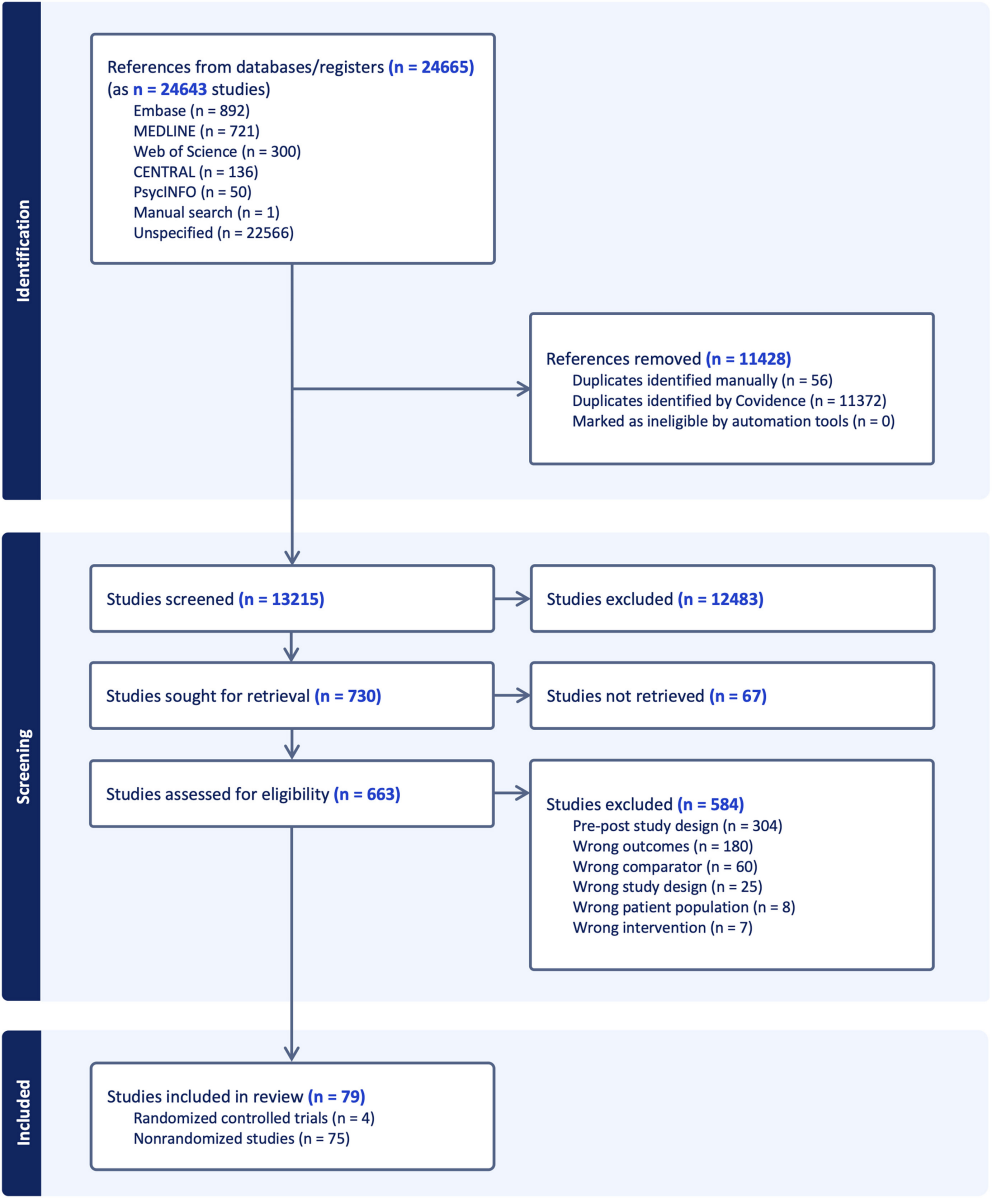
Title: Flow of Studies. Caption: PRISMA (Preferred Reporting Items for Systematic Reviews and Meta‐Analyses) flowchart showing the flow of studies throughout the systematic review process.

The searches generated 24,665 abstracts. There were 13,215 titles and abstracts screened, and 730 had full texts retrieved. Of these, 79 were eligible (Figure 1). One paper was written in Portuguese, and the rest were in English language.

Characteristics and risk of bias of these 79 studies are shown in Table 1. Seventy‐five were NRS, and 4 were RCTs with 242 total participants. Five were conference abstracts. Seventy‐one studies were conducted in high‐income countries. Duration of follow‐up ranged from 3 to 240 months (mean = 37 months). Surgical techniques included sleeve gastrectomy, gastric bypass, biliopancreatic diversion with duodenal switch, vertical gastroplasty, DJBL, and gastric banding. A passive control group was used in 70% of studies.

**TABLE 1:** Characteristics of included studies.

Author, year	Study design	Follow‐up, months	Bariatric procedure, *N*	Control description, *N*	Country, income type	Baseline psychiatric disorder/treatment use, proportion	Mean age, years		Proportion female		Overall risk of bias
							Surgery	Control	Surgery	Control	
		Randomized controlled trials									
Cohen, 2022^93^	RCT	60	RYGB, 51	MWM, 49	Brazil, middle	NR	53	53	45%	45%	High
Jarvholm, 2023^46^	RCT	24	RYGB + SG, 25	Lifestyle, 25	Sweden, high	33%	16	16	74%	74%	Some concerns
Koschker, 2023^47^	RCT	12	RYGB, 22	Lifestyle, 24	Germany, high	NR	43	43	85%	85%	High
		Non‐randomized studies									
Ahmadzad‐Asl, 2022^94^	PCS	3	NR, 40	Wait‐list, 30	Iran, middle	16%	39	41	88%	93%	Critical
Ahmed, 2013^95^	RCS	29	RYGB + SG + GB, 144	No intervention, 1440	United States, high	100%	44	45	89%	89%	Serious
Alosco, 2014^96^	PCS	24	RYGB + GB, 63	No intervention, 23	United States, high	NR	42	41	91%	96%	Critical
Arhi, 2021^97^	RCS	120	NR, 3543	No intervention, 15480	United Kingdom, high	43%	NR	NR	79%	80%	Serious
Bartsch, 2016^98^	XS	120	RYGB + SG, 73	Wait‐list, 71	Germany, high	NR	41	41	78%	78%	Critical
Becerra, 2022^99^	RCS	12–36	RYGB + SG, 56661	No intervention, 56661	United States, high	19%	48	48	81%	81%	Serious
Booth, 2015^100^	RCS	24–84	RYGB + SG + GB, 3045	No intervention, 3045	United Kingdom, high	NR	46	44	79%	83%	Serious
Boulon, 2022^71^	PCS	36	RYGB + SG, 15	MWM, 17	NR	NR	38	47	87%	77%	Critical
Bramming, 2021^101^	RCS	83	RYGB + SG + GB, 12251	No intervention, 16005	Denmark, high	NR	43	43	72%	74%	Serious
Buddeberg‐Fischer, 2006^102^	PCS	38	RYGB + GB, 63	No intervention, 30	Switzerland, high	NR	44	NR	NR	NR	Critical
Butt, 2023^77^	RCS	24	RYGB + SG + GB, 38525	No intervention, NR	United States, high	NR	NR	NR	NR	NR	Critical
Chandarana, 1988^73^	XS	20	GB, 31	Wait‐list, 31	NR	NR	38	37	90%	84%	Critical
Conceicao, 2014^49^	XS	24	RYGB + SG + GB, 163	Wait‐list, 176	Portugal, high	NR	42	42	89%	89%	Critical
Conceicao, 2020^103^	XS	18–36	RYGB + SG, 65	Wait‐list, 65	Portugal, high	NR	NR	NR	91%	85%	Critical
Decker, 2022^104^	PCS	72	RYGB + SG + GB, 83	Lifestyle, 139	United States, high	46%	17	16	80%	82%	Serious
De Lourdes, 2022^105^	XS	12	RYGB + SG + GB, 131	Wait‐list, 163	Portugal, high	NR	43	41	89%	80%	Critical
De Oliveira, 2009^106^	XS	6–33	NR, 33	Wait‐list, 32	Brazil, middle	NR	31–40	31–40	88%	97%	Critical
De Zwaan, 2014^107^	XS	1–15.5 (years)	SG + GB, 314	Wait‐list, 79	Germany, high	NR	48	42	85%	85%	Critical
Dischinger, 2023^108^	XS	46–58	RYGB + SG, 20	No intervention, 22	Germany, high	NR	39 (RYGB), 34 (SG)^a^	37, 34^a^	50%	64%	Critical
Faulconbridge, 2013^64^	PCS	12	RYGB + GB, 36	Lifestyle, 49	United States, high	NR	47	44	72%	80%	Critical
Figura, 2017^72^	PCS	19	SG, 62	Lifestyle, 40	Germany, high	NR	46	51	71%	78%	Critical
Fisher, 2018^67^	RCS	24	RYGB + SG, 31	NR, 828	United States, high	NR	49	46	94%	82%	Critical
Fu, 2023^109^	RCS	112	NR, 110	No intervention, 321	China, high	NR	53	53	79%	76%	Serious
Georgiadou, 2014^110^	XS	14	RYGB, 50	Wait‐list, 50	Germany, Turkey, Russia, and Poland, high and middle	NR	42	42	86%	86%	Serious
Gouselard, 2022^111^	RCS	36	NR, 1984	No intervention, 5952	France, high	6%	22	22	92%	72%	Critical
Greco, 2021^74^	PCS	3	RYGB + SG, 27	MWM, 25	Canada, high	NR	41	44	100%	100%	Critical
Herpertz, 2015^65^	PCS	24	GB, 152	Lifestyle + no intervention, 249	Germany, high	NR	39	42	67%	73%	Critical
Hung, 2023^112^	RCS	55	RYGB + SG, 3813	No intervention, 32015	United States, high	41%	53	54	24%	21%	Serious
Jakobsen, 2018^113^	PCS	24–84	RYGB, 9 + SG, 32	Lifestyle, 956	Norway, high	40%	44	46	69%	64%	Critical
Jarvholm, 2020^114^	PCS	60	NR, 81	Lifestyle, 80	Sweden, high	19%	17	16	65%	56%	Serious
Kim, 2023^115^	RCS	120	NR, 17026	No intervention, 34052	United States, high	41%	42	42	78%	78%	Serious
Klemenic, 2021^116^	PCS	12	DJBL, 19	Lifestyle, 26	Slovenia, high	NR	17	16	63%	50%	Critical
Kontinnen, 2015^117^	PCS	12–120	RYGB + GB, 2010	No intervention, 1916	Sweden, high	NR	47	49	71%	71%	Serious
Kontinnen, 2021^118^	RCS	12	RYGB + SG, 2007	No intervention, 2040	Sweden, high	11%	47	49	71%	71%	Serious
Kovacs, 2017^79^	RCS	48	NR, 12612	No intervention, 9839	Denmark, high	NR	42	NR	75%	87%	Critical
Larsen, 2004^119^	XS	24	GB, 157	Wait‐list, 93	Netherlands, high	NR	40	39	92%	83%	Critical
Legenbauer, 2020^120^	PCS	108	SG + GB, 78	MWM + no intervention, 124	Germany, high	NR	46	51	69%	73%	Critical
Liakopoulos, 2019^121^	RCS	108	RYGB, 5321	No intervention, 5321	Sweden, Europe, high	6%	49	47	61%	64%	Serious
Liu, 2022^122^	XS	NR	NR, 89	No intervention, 1082	United States, high	NR	NR	NR	NR	NR	Critical
Lu, 2018^123^	RCS	24–120	RYGB + SG + GB, 2302	No intervention, 6493	Taiwan, high	NR	32	33	62%	62%	Serious
Mabey, 2021^124^	PCS	144	RYGB, 131	No intervention, 205	United States, high	NR	56	60	83%	81%	Critical
Maciejewski, 2020^125^	RCS	24–96	RYGB + SG, 2608	No intervention, 24232	United States, high	44%	53	54	27%	NR	Serious
Mackey, 2018^81^	PCS	3–4	SG, 12	Lifestyle + wait‐list, 19	United States, high	23%	17	16	58%	71%	Critical
Mahmud, 2023^126^	RCS	60	RYGB + SG + GB, 6330	Lifestyle, 1364	United States, high	NR	53 (RYGB), 52 (SG), 55 (GB)^a^	59	30% (RYGB), 33% (SG) 25% (GB)^a^	14%	Serious
Mellinger, 2021^78^	RCS	44	RYGB + SG + GB, 403220	No intervention, 6612371	United States, high	NR	44	NR	80%	NR	Critical
Mirijello, 2015^127^	PCS	44	BPD/DS, 26	Wait‐list, 20	Italy, high	NR	41	44	85%	50%	Serious
Neovius, 2018^128^	RCS	96	VBG + GB, 22264	Lifestyle, 18,199	Sweden, high	31%	47 (VBG), 41 (GB)^a^	49, 42^a^	71% (VBG), 79% (GB)^a^	71%, 79%^a^	Moderate
Nickel, 2007^129^	PCS	72	GB, 22	No intervention, 35	Germany, high	NR	38	40	100%	100%	Critical
Nielsen, 2017^130^	XS	11	RYGB + SG, 128	Wait‐list, 120	Germany, high	NR	42	41	79%	79%	Serious
Noli, 2010^131^	XS	12	BPD/DS, 133	Wait‐list, 150	Italy, high	NR	43	42	63%	65%	Critical
Paczkowska, 2022^66^	PCS	12	RYGB + SG + GB, 155	MWM + lifestyle, 409	Poland, Germany, high	NR	46 (Germany), 45 (Poland)^b^		75% (Germany), 77% (Poland)^b^		Critical
Pasi, 2023^68^	PCS	6	RYGB + SG, 46	MWM, 17	Switzerland, high	100%	44	41	76%	82%	Critical
Prehn, 2020^80^	PCS	6	RYGB + GB, 40	Wait‐list, 29	Germany, high	NR	46	45	70%	55%	Critical
Ribeiro, 2022^132^	PCS	6	SG, 21	Lifestyle, 21	Portugal, high	NR	41	41	100%	100%	Critical
Schowalter, 2008^133^	PCS	60–84	GB, 128	Lifestyle + no intervention, 120	Germany, high	NR	38	38	82%	83%	Critical
Shariati, 2023^134^	PCS	4	RYGB + SG, 24	Wait‐list, 49	Iran, middle	NR	63	64	71%	71%	Critical
Smith, 2022^135^	RCS	24–60	941 (RYGB‐D) + 772 (SG‐D) + 1275 (RYGB‐ND), + 952 (SG‐ND)^a^	18,114 + 6942 no intervention‐D, 11895 + 9044 no intervention‐ND^a^	United States, high	42%	52 (RYGB‐D), 53 (SG‐D), 53 (RYGB‐ND), 53 (SG‐ND)^a^	53 + 53 (no intervention‐D), 54 + 54 (no intervention‐ND)^a^	30% (RYGB‐D), 32% (SG‐D), 19% (RYGB‐ND), 19% (SG‐ND)^a^	26% + 29% (no intervention‐D), 16% + 17% (no intervention‐ND)^a^	Moderate
Stenberg, 2022^136^	PCS	24–60	SG, 1216	Lifestyle, 2432	Sweden, high	48%	42	43	90%	90%	Moderate
Svensson, 2013^137^	PCS	168	265 (GB) + 376 (banding) + 1369 (VBG)^a^	Lifestyle + no intervention, 2037	Sweden, high	NR	47 (GB), 48 (banding), 47 (VBG)^a^	49	71% (GB), 69% (banding), 71% (VBG)^a^	71%	Serious
Svensson, 2023^138^	RCS	48	GB, 28204	No intervention, 40827	Sweden, high	35%	41	43	76%	69%	Serious
Svensson, 2023^139^	PCS	286	264 (GB) + 373 (banding) + 1353 (VBG)^a^	No intervention, 2030	Sweden, high	16%	47 (GB) + 48 (banding) + 47 (VBG)^a^	49	72% (GB) + 71% (banding) + 71% (VBG)^a^	71%	Serious
Tan, 2021^140^	NRCT	12	RYGB + SG, 55	MWM, 85	Singapore, high	26%	45	50	64%	59%	Critical
Tinos, 2022^70^	PCS	12	NR, 46	No intervention, 43	Brazil, middle	NR	NR	NR	NR	NR	Critical
Vafa, 2023^141^	PCS	12	RYGB, 25	No intervention, 29	Iran, middle	22%	39	36	72%	76%	Critical
Vangoitsenhoven, 2016^63^	PCS	84	RYGB, 23	Wait‐list, 23	Belgium, high	NR	49	49	74%	74%	Serious
Wallen, 2023^142^	PCS	24–96	RYGB + SG, 30359	Lifestyle, 21,356	Sweden, high	30%	40	41	79%	79%	Serious
Walsh, 2018^69^	PCS	6	RYGB + SG + GB, 29	Wait‐list, 16	Australia, high	NR	45	45	86%	81%	Critical
Yuan, 2019^143^	RCS	25	RYGB + SG + GB + BPD/DS, 64090	NR, 713050	United States, high	NR	46	53	71%	54%	Serious
Zeller, 2017^144^	PCS	24	RYGB + SG + GB, 216	Lifestyle, 79	United States, high	NR	17	16	75%	82%	Critical
Zeller, 2020^145^	PCS	24	RYGB + SG + GB, 153	Lifestyle, 70	United States, high	19%	17	16	79%	80%	Critical
Zeller, 2023^146^	PCS	72	RYGB + SG + GB, 139	Lifestyle, 83	United States, high	NR	17	16	80%	85%	Critical
		Conference abstracts									
Matsumoto, 2017^82^	PCS	6	NR, 4	No intervention, 5	NR	NR	NR	NR	NR	NR	Critical
Oh, 2016^76^	RCS	NR	NR	No intervention	United States, high	NR	NR	NR	NR	NR	Critical
			Total = 986,120								
Pinto, 2009^147^	XS	24	NR, 60	Wait‐list, 60	Brazil, middle	NR	NR	NR	100%	100%	Critical
Scholtz, 2010^148^	XS	6	RYGB, 16	Wait‐list, 12	United Kingdom, high	NR	NR	NR	88%	92%	Critical
Vargas, 2022^75^	RCT	12	SG, 26	Lifestyle, 20	United States, high	NR	NR	NR	NR	NR	High

Abbreviations: BPD/DS, biliopancreatic diversion with duodenal switch; DJBL, duodenal‐jujunal bypass liner; GB, gastric bypass; MWM, medical weight management; NR, not reported; NRCT, non‐randomized controlled trial; PCS, prospective cohort study; RCS, retrospective cohort study; RCT randomized controlled trial; RYGB, Roux‐en‐Y Gastric Bypass; RYGB‐D, Roux‐en‐Y Gastric Bypass depressed at baseline; RYGB‐ND Roux‐en‐Y Gastric Bypass not depressed at baseline; SG, sleeve gastrectomy; SG‐D, sleeve gastrectomy depressed at baseline; SG‐ND, sleeve gastrectomy not depressed at baseline; VBG, vertical banded gastroplasty; XS, cross‐sectional study.

^a^ Reported separately by treatment type (otherwise presented together).

^b^ Reported separately by country (otherwise presented together).

Of 8,402,919 people with obesity, 732,149 underweight MBS and 7,670,770 did not. Mean age of participants was 41 and 63% were female. Of the 38 studies that defined surgical eligibility by BMI cut‐off, 23% used BMI > 30 kg/m^2^, 74% used > 35 kg/m^2^, and 8% used > 40 kg/m^2^. Only 25 studies reported psychiatric history at baseline, and of these, 32% of participants had at least 1 disorder or were taking psychiatric treatment.

### 3.1 Depressive Symptoms

**FIGURE 2:**
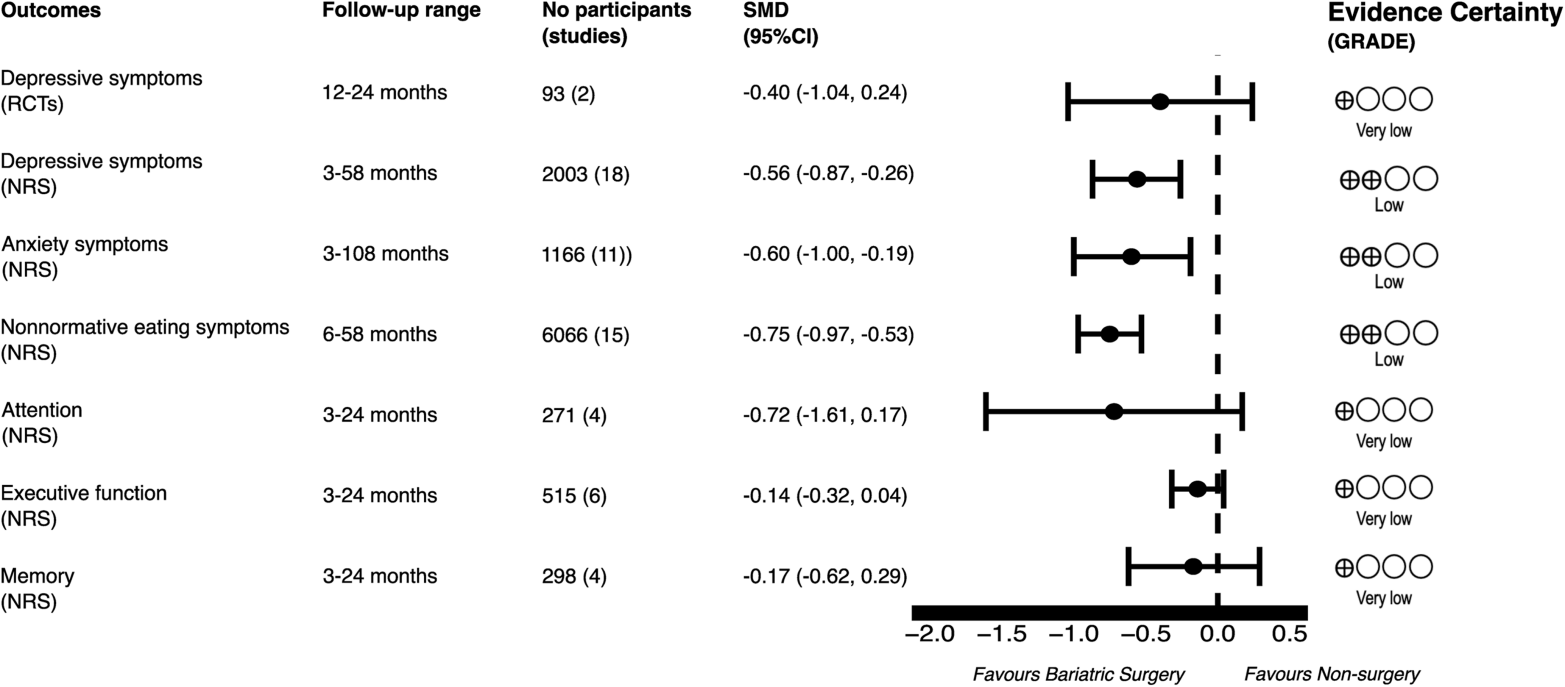
Title: Continuous Outcomes Meta‐Analysis Summary. Caption: Forest plot summary showing effect sizes between MBS and nonsurgical control groups, incorporating both non‐randomized studies (NRS) and randomized controlled trials (RCTs). SMD = standardized mean difference.

From a meta‐analysis of 93 people from 2 RCTs, MBS may improve depressive symptoms over 12–24 months (SMD = −0.40, 95% CI −1.04, 0.24; Table 2, Figure 2), but the evidence is very uncertain. In a meta‐analysis of 2072 people from 18 NRS, MBS may improve depressive symptoms over 3–58 months (SMD = −0.56, 95% CI −0.87, −0.26; low certainty; Table 2, Figure 2). Removing studies at critical risk of bias, with passive comparators, and that used a DJBL intervention resulted in similar effect sizes. In 5 NRS ineligible for meta‐analysis, evidence of benefit was also found in at least 1 study (*p* = 0.03) [59, 60, 61, 62, 63]. Two additional NRS were consistent with the direction of effect in the meta‐analyses [64, 65].

**TABLE 2:** Summary of findings for main outcomes.

Outcomes	Anticipated absolute effects* (95% CI)		Relative effect (95% CI)	No. of participants (studies)	Certainty of the evidence (GRADE)	Comments
	Risk with no intervention	Risk with bariatric surgery				
Depressive symptoms follow‐up: range 12 to 24 months	—	SMD 0.4 lower (1.04 lower to 0.24 higher)	—	93 (2 RCTs)	⨁ ◯ ◯ ◯ Very low^a^, ^b^	MBS may improve depressive symptoms, but the evidence is very uncertain.
Depressive symptoms follow‐up: range 3 to 58 months	—	SMD 0.56 SD lower (0.87 lower to 0.26 lower)	—	2003 (18 NRS)	⨁ ⨁ ◯ ◯ Low^c^	MBS may improve depressive symptoms.
Anxiety symptoms follow‐up: range 3 to 108 months	—	SMD 0.6 lower (1 lower to 0.19 lower)	—	1166 (11 NRS)	⨁ ⨁ ◯ ◯ Low^c^	MBS may improve anxiety symptoms.
Non‐normative eating symptoms follow‐up: range 6 to 58 months	—	SMD 0.75 SD lower (0.97 lower to 0.53 lower)	—	6066 (15 NRS)	⨁ ⨁ ◯ ◯ Low^c^	MBS may improve non‐normative eating symptoms.
Substance use disorder proportion ≤ 2 years of follow‐up	4 per 100	4 per 100 (2 to 5)	**RR 0.97** (0.64 to 1.46)	151,431 (5 NRS)	⨁ ⨁ ⨁ ◯ Moderate^d^	MBS probably does not reduce substance use disorders within 2 years of follow‐up.
Substance use disorder proportion > 2 years of follow‐up	4 per 100	8 per 100 (5 to 13)	**RR 2.13** (1.33 to 3.42)	193,959 (8 NRS)	⨁ ⨁ ◯ ◯ Low^d^, ^e^	MBS may slightly increase substance use disorders after 2 years of follow‐up.
Suicide death proportion follow‐up: range 4 to 21 years	2 per 1000	3 per 1000 (2 to 5)	**RR 1.86** (1.07 to 3.21)	167,042 (5 NRS)	⨁ ◯ ◯ ◯ Very low^c^, ^f^	MBS may slightly increase suicide deaths, but the evidence is very uncertain.
Attention follow‐up: range 3 to 24 months	—	SMD 0.72 higher (0.17 lower to 1.61 higher)	—	271 (4 NRS)	⨁ ◯ ◯ ◯ Very low^c^, ^g^	MBS may improve cognitive performance in attention, but the evidence is very uncertain.
Executive function follow‐up: range 3 to 24 months	—	SMD 0.14 higher (0.04 lower to 0.32 higher)	—	515 (6 NRS)	⨁ ◯ ◯ ◯ Very low^c^, ^h^	MBS may have little to no effect on cognitive performance in executive function, but the evidence is very uncertain.
Memory follow‐up: range 3 to 24 months	—	SMD 0.17 higher (0.29 lower to 0.62 higher)	—	298 (4 NRS)	⨁ ◯ ◯ ◯ Very low^c^, ^g^	MBS may have little to no effect on cognitive performance in memory, but the evidence is very uncertain.

*Note:* **GRADE Working Group grades of evidence. High certainty:** We are very confident that the true effect lies close to that of the estimate of the effect. **Moderate certainty:** We are moderately confident in the effect estimate: The true effect is likely to be close to the estimate of the effect, but there is a possibility that it is substantially different. **Low certainty:** Our confidence in the effect estimate is limited: The true effect may be substantially different from the estimate of the effect. **Very low certainty:** We have very little confidence in the effect estimate: The true effect is likely to be substantially different from the estimate of effect.

Abbreviations: CI, confidence interval; MD, mean difference; RR, risk ratio; SMD, standardized mean difference.

* The risk in the intervention group (and its 95% CI) is based on the assumed risk in the comparison group and the relative effect of the intervention (and its 95% CI).

^a^ Around half of the studies in this meta‐analysis are at high risk of bias.

^b^ The 95% CI crosses a large reduction (SMD < −0.8) and a small increase (SMD > 0.2). Inconsistency is likely driving some of this imprecision, though it did not downgrade separately for inconsistency.

^c^ All studies are at either serious or critical risk of bias overall.

^d^ Most studies are at either serious or critical risk of bias overall.

^e^ Considerable heterogeneity (I^2^ = 99%, many studies have substantially different effect from the point estimate) that is likely leading to imprecision (the 95% CI crosses the threshold of a difference in 2/100 people).

^f^ The 95% CI crosses the threshold of a difference in 1/1000 people. Inconsistency is likely driving some of the imprecision, though did not downgrade separately for inconsistency.

^g^ The 95% CI crosses the threshold for appreciable benefit (SMD > 0.2). Inconsistency is likely driving some of this imprecision, though did not downgrade separately for inconsistency.

^h^ The 95% CI crosses the threshold for appreciable benefit (SMD > 0.2).

### 3.2 Anxiety Symptoms

Meta‐analytically, in 1166 people from 11 NRS, MBS may improve anxiety symptoms over 3–108 months (SMD = −0.60, 95% CI −1.00, −0.19; low certainty; Table 2, Figure 2). Results were similar in sensitivity analyses when studies at critical risk of bias and that used a DJBL intervention were removed, but not when those with passive comparators were (SMD = −0.03, 95% CI −0.33, 0.27). No evidence of benefit was found on anxiety symptoms in 2 NRS ineligible for meta‐analysis (*p* = 0.61) and in 2 additional NRS and a RCT [45, 59, 61, 62, 66].

### 3.3 Non‐Normative Eating Symptoms

From a meta‐analysis of 6066 people from 15 NRS, MBS may improve non‐normative eating symptoms over 6–58 months (SMD = −0.75, 95% CI −0.97, −0.53; low certainty; Table 2, Figure 2). Symptoms included in this composite included disordered eating (4 studies), loss of control over eating (4 studies), emotional eating (4 studies), binge eating (1 study), weight preoccupation (1 study), and eating concern (1 study). Sensitivity analyses showed consistent results. Narrative synthesis did not consistently align with the meta‐analysis. In 5 NRS ineligible for meta‐analysis, there was no evidence of benefit on non‐normative eating symptoms (*p* = 0.17), nor was there in another NRS or RCT conference abstract [59, 60, 67, 68, 69, 70, 71]. However, an RCT found a large evidence of benefit, consistent with the meta‐analysis [45].

### 3.4 Substance Use Disorders

**FIGURE 3:**
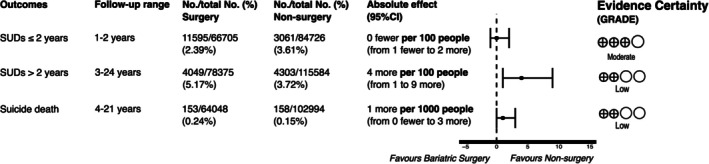
Title: Dichotomous Outcomes Meta‐Analysis Summary. Caption: Forest plot summary showing effect sizes between MBS and nonsurgical control groups, of non‐randomized studies. SUDs = substance use disorders; RR = risk ratio. Suicide death denominator is out of 1000 people.

Meta‐analytically, in 271,240 people from 10 NRS, MBS may slightly increase substance use disorders (RR = 1.66, 95% CI 1.00–2.78; 2/100 more people, 95% CI from 0 fewer to 5 more); however, heterogeneity was high (I^2^ = 99%) and there was a significant subgroup effect for duration of follow‐up (test for difference *p* = 0.01). Within 2 years of follow‐up, MBS probably does not reduce substance use disorders (RR = 0.97, 95% CI 0.64–1.46; 0/100 fewer people, 95% CI from 13 fewer to 17 more; NNT = 83; moderate certainty), but may slightly increase substance use disorders after 2 years (RR = 2.13, 1.33–3.42; 4/100 more, 95% CI from 1 to 9 more; NNH = 67; low certainty; Table 2, Figure 3). Substances included in this composite included alcohol (6 studies), any substance (1 study), and non‐alcohol substance (1 study) use disorders. Sensitivity analyses showed consistent results. In 4 NRS ineligible for meta‐analysis, there was no difference in substance use disorders (*p* = 0.80), however, a RCT aligned with the latter subgroup analysis suggesting evidence of harm [45, 72, 73, 74, 75].

### 3.5 Suicide Death

From a meta‐analysis of 167,042 people from 5 NRS, MBS may slightly increase suicide deaths over 4–21 years (RR = 1.86, 95% CI 1.07–3.21; 1/1000 more people, 95% CI 0 to 3 more; NNH = 1111; Table 2, Figure 3), but the evidence is very uncertain. Results were consistent in a sensitivity analysis removing passive comparator studies.

### 3.6 Cognitive Performance

Several meta‐analyses on cognitive performance were conducted. In 271 people from 4 NRS, MBS may improve cognitive performance in attention over 3–24 months (SMD = −0.72, 95% CI −1.61, 0.17; Table 2, Figure 2), but the evidence is very uncertain. No sensitivity analyses were possible. An NRS ineligible for meta‐analysis did not align with this finding [76].

In 515 people from 6 NRS, MBS may have little to no effect on cognitive performance in executive function over 3–24 months (SMD = −0.14, 95% CI −0.32, 0.04; Table 2, Figure 2), but the evidence is very uncertain. Results were sustained in a sensitivity analysis removing critical risk of bias studies. Three NRS ineligible for meta‐analysis were consistent with this finding [76, 77, 78].

In 298 people from 4 NRS, MBS may have little to no effect on cognitive performance in memory over 3–24 months (SMD = ‐0.17, 95% CI −0.62, 0.29; Table 2, Figure 2), but the evidence is very uncertain. Results were sustained in a sensitivity analysis removing critical risk of bias studies. Two NRS ineligible for meta‐analysis were consistent with this finding [76, 77].

### 3.7 Other Outcomes

MBS may not change depressive disorders (5 NRS; RR = 1.07, 95% CI 0.94–1.21; 1/100 more people, 95% CI from 1 fewer to 2 more; low certainty), dementia (5 NRS; RR = 0.89, 95% CI 0.39–2.01; 0/100 fewer people, from 0 fewer to 1 more; low certainty), psychiatric hospitalizations (5 NRS; RR = 1.17, 95% CI 0.98–1.40; 1/100 more, 95% CI from 0 fewer to 2 more; low certainty), AUDIT‐C symptoms (1 NRS; MD = 0.30, 95% CI 0.18–0.42; low certainty), and suicidal ideation/nonfatal self‐harm behavior within 2 years (2 NRS; RR = 0.45, 95% CI 0.39–0.52; 1/100 fewer people, 95% CI from 1 to 0 fewer; low certainty), and probably does not change suicidal ideation/nonfatal self‐harm behavior after 2 years (4 NRS; RR = 1.02, 95% CI 0.93–1.11; 0/100 fewer people, 95% CI from 0 fewer to 0 more; moderate certainty). MBS may slightly increase anxiety disorders (5 NRS; RR = 1.16, 95% CI 1.13–1.19; 2/100 more people, 95% CI from 2 to 3 more; low certainty) and eating disorders (10 NRS; RR = 5.32, 95% CI 4.84–5.86; 4/100 more people, from 3 to 4 more; very low certainty), and probably slightly increases psychiatric treatment use (9 NRS; RR = 1.22, 95% CI 1.08–1.38; 9/100 more, 95% CI from 3 to 15 more; moderate certainty). MBS may result in a large increase in psychiatric disorder diagnoses (3 NRS; RR = 1.68, 95% CI 1.34–2.10; 32/100 more, 95% CI from 16 to 51 more; low certainty). See Tables S2, S3 and Results S1 in the Supporting Information for details.

## 4 Discussion

This paper presented a comprehensive systematic review and meta‐analysis of the effects of MBS on psychiatric and cognitive functioning in people with obesity. The main results suggest that, compared to nonsurgical options, MBS may improve some aspects of psychiatric and cognitive functioning (depressive, anxiety, and non‐normative eating symptoms, and cognitive performance in attention) while slightly worsening other aspects of functioning more than 2 years after surgery (substance use disorders and suicide death). However, these results must be interpreted cautiously as the strength of evidence is low to very low for most outcomes. In the implementation of GRADE, evidence was most often downgraded because of the risk of bias in NRS (mostly due to the potential for confounding bias), as well as imprecision around effect estimates (i.e., statistical uncertainty). Therefore, additional high‐quality studies with large sample sizes are needed.

Results suggest that MBS may improve depressive and anxiety symptoms, compared to nonsurgical options, though certainty in these findings is low to very low. This paper contributes the first meta‐analysis of RCTs, showing that the effect on depression may be meaningful but smaller than prior estimates; however, additional RCTs with larger sample sizes are needed to improve confidence in this finding. Previous meta‐analyses of NRS also found benefit for depression and anxiety, but within‐groups studies were often pooled, which can introduce maturation (e.g., spontaneous symptom improvement that is common in depression) and/or survivor bias (e.g., those with the highest pre‐operative anxiety symptoms may be less likely to attend post‐operative visits), which could partially explain this difference [25, 26, 79]. A sensitivity analysis including only studies with an active control group did not sustain the positive effect between MBS and anxiety symptoms. This suggests that anxiety symptom change could be related to the amount of weight reduction, since active nonsurgical interventions can substantially reduce weight, whereas passive groups like surgical waitlists may not [20, 80].

To the authors' knowledge, this paper adds the first meta‐analytical evidence of non‐normative eating after MBS compared to nonsurgical controls, showing a large improvement in symptoms, in keeping with a past narrative review [28]. In contrast, this paper found that MBS may slightly increase the prevalence of eating disorders, though there is very low certainty in this outcome and the effect was not sustained in a sensitivity analysis including only studies with active control groups. While non‐normative eating may worsens after MBS compared with nonsurgical options, it is also possible that surgical groups are followed more closely and so may be more likely to have eating disorders diagnosed. Overall, this is an important outcome that needs to be evaluated further because non‐normative eating is associated with reduced quality of life in people with obesity and improving these symptoms is a reason why some people decide to pursue MBS [81]. The low to very low evidence certainty and discordant findings necessitate future high‐quality research to clarify the effect of MBS on non‐normative eating behavior.

Despite evidence that obesity is linked with reduced cognitive performance and the development of dementia over time, this paper did not find convincing evidence of an association between MBS and the cognitive outcomes examined [82]. While very low certainty evidence suggests that MBS may improve cognitive performance in attention, little to no change was found in domains of memory and executive function or in dementia prevalence. Findings align with a recent meta‐analysis of within‐groups studies that found improved attention after MBS, but contrast with findings of improved memory and executive function 3 months after surgery [83]. This contrast could be due to a practice effect in within‐groups studies, especially given the brief duration between tests. In the present meta‐analyses, studies were also of short duration (usually < 6 months), which may not be long enough for cognitive changes to occur post‐operatively compared to nonsurgical controls. Participants were also young, and there were too few participants studied to be certain in results. The effect of MBS on cognitive functioning remains unclear and warrants further investigation with adequately powered, methodologically rigorous studies with longer duration of follow‐up (ideally greater than 2 years).

In line with existing work, this paper found MBS slightly increased substance use disorders and suicide death after more than 2 years; however, there is low and very low certainty in these outcomes, respectively [35, 84]. Evidence could be strengthened by further high‐quality studies in relevant subgroups (e.g., different substances, with and without substance use disorders at baseline) for substance use disorders and by including more participants in studies on suicide death. Suicide is rare (baseline rate ~1/10,000) and so it may not be possible to reliably assess in RCTs that may be less prone to bias than NRS, and it is generally difficult to determine whether someone died from suicide as it can be misreported [85, 86]. Due to the low baseline rate in individual studies, the absolute increase in suicide death in the current meta‐analysis was very small (i.e., 1 more person out of 1000 may die by suicide after receiving MBS, NNH = 1111). Findings that MBS may not effect suicidal ideation and self‐harm behavior is not necessarily contradictory, as suicide cannot be reliably predicted and many people who die by suicide do not have a history of self‐harm [87]. Mechanisms to explain the possible heightened risk of suicide after MBS are unknown, however, increased sensitivity to substances and substance use disorders could contribute by influencing impulsive behavior [88]. For some, initial health improvements may not persist, possibly leading to self‐blame and regret as expectations of surgery were not met. Environmental factors that are associated with suicide may also affect people post‐operatively, such as persistent discrimination and relationship breakdown [86].

### 4.1 Limitations

While 6 databases were searched, it is possible that individual studies contained in other databases were missed. Papers examining revisional surgeries were excluded; therefore, this study cannot address associations between psychiatric and cognitive functioning and these interventions. While a range of outcomes were examined, not all aspects of psychiatric and cognitive functioning were feasible to combine in a single paper (e.g., post‐traumatic stress, neuroimaging findings, language skills). While 35 authors were contacted twice for missing data, only 14 responded, and there is uncertainty about the impact of these missing data on findings. Outcomes were pooled broadly to provide the strongest overall effect estimates, however, this decision reduced nuance and could have increased inconsistency or imprecision. For example, while overall effect estimates for non‐normative eating and substance use are provided, this review does not allow for conclusions to be drawn about specific symptoms (e.g., binge eating vs. emotional eating) or substances (e.g., cannabis vs. alcohol). Eating disorders were also defined using study‐defined cut‐offs for non‐normative eating behavior, and not necessarily diagnosed eating disorders, which may have overestimated their prevalence. Surgical techniques were pooled broadly and there are differences in efficacy and adverse effects that could impact the brain differentially; however, a sensitivity analysis removing DJBL studies did not change results [20]. Since included NRS varied in their study designs, methods to address confounding, and number of potential confounders controlled for, all NRS were pooled together, using their unadjusted results. This decision may have introduced additional biases in the effect estimates. To reduce the potential for bias, only between‐groups studies were included and just 1 subgroup analysis was conducted; these decisions were a diversion from the original protocol. Regarding the evidence included in the review, the main limitation is that there is low to very low certainty in most outcomes.

### 4.2 Conclusions

Evidence suggests that in people with obesity, MBS may improve depression, anxiety, non‐normative eating, and cognitive performance in attention, but also slightly increase substance use disorders and suicide death in the longer term. However, there was low to very low certainty in most findings. Despite the reduced certainty, these findings reflect the best available evidence and can be considered by people who are contemplating or have received MBS, their families, healthcare workers, as well as guideline developers in the field of obesity. More methodologically rigorous and adequately powered studies of MBS compared to best practice medical weight management are recommended to strengthen confidence in the effects of MBS on psychiatric and cognitive functioning. This could be achieved by incorporating psychiatric and cognitive measures into MBS RCTs as secondary outcomes or embedded sub‐studies, and by adjusting for important confounding variables in high‐quality NRS.

## Conflicts of Interest

The authors declare no conflicts of interest.

## Supporting information


**Data S1.** Medline database search strategy.
**Table S1.** Hierarchy of outcome selection.
**Table S2.** Evidence profile for all outcomes.
**Table S3.** Summary of findings for other outcomes.
**Data S2.** Meta‐analyses and narrative syntheses for other outcomes.
