# Supplementary material for: Psychiatric and Cognitive Functioning After Metabolic and Bariatric Surgery: A Systematic Review and Meta‐Analysis
Source: Obes Rev. 2025 Jun 22;26(11):e13968. doi: 10.1111/obr.13968 (PMC12531740; doi:10.1111/obr.13968)
Supplement: Supplementary file 1 — Data S1. Medline database search strategy. Table S1. Hierarchy of outcome selection. Table S2. Evidence profile for all outcomes. Table S3. Summary of findings for other outcomes. Data S2. Meta‐analyses and narrative syntheses for other outcomes. [file OBR-26-e13968-s001.pdf]

Supplementary Information

Title: Psychiatric and cognitive functioning after metabolic and bariatric surgery: a systematic review and meta-analysis

Authors and affiliations:
Emma A van Reekum MD[1,2,3], emma.vanreekum@medportal.ca
Michael Darcy MD[4], michael.darcy@medportal.ca
Jaslyn Drage BSc[1], dragej@mcmaster.ca
Joshua Xu MD PhD[5], joshua.xu@medportal.ca
Kimberly Ng MD[1], kim.ng@medportal.ca
Benjamin Forestell MD[6], ben.forestell@medportal.ca
Nancy Santesso PhD[2], santesna@mcmaster.ca
Raed A Joundi MD PhD[3], Raed.Joundi@phri.ca
Jorge Wong MD PhD[3], Jorge.Wong@phri.ca
Aristithes Doumouras MD MpH[7], aristithes.doumouras@medportal.ca
Valerie H Taylor MD PhD[8], valerie.taylor3@albertahealthservices.ca
Salim Yusuf* MD PhD[2,3], yusufs@mcmaster.ca
Ryan Van Lieshout* MD PhD[1,2], vanlierj@mcmaster.ca
*co-senior authors

1.Department of Psychiatry and Behavioural Neurosciences, Faculty of Health Sciences, McMaster University, Hamilton, Ontario, Canada
2. Department of Health Research Methods, Evidence, and Impact, Faculty of Health Sciences, McMaster University, Hamilton, Ontario, Canada
3. Population Health Research Institute, McMaster University and Hamilton Health Sciences, Hamilton, Ontario, Canada
4. Department of Psychiatry, University of Toronto, Toronto, Ontario, Canada
5. Department of Surgery, University of Calgary, Calgary, Alberta, Canada
6. Emergency Medicine, Faculty of Health Sciences, McMaster University, Hamilton, Ontario, Canada
7. Department of Surgery, Faculty of Health Sciences, McMaster University, Hamilton, Ontario, Canada
8. Department of Psychiatry, University of Calgary, Calgary, Alberta, Canada

Title	Page
**Methods S1.** Medline Database Search Strategy	3
**Table S1.** Hierarchy of Outcome Selection	6
**Table S2:** Evidence Profile for All Outcomes	7
**Table S3:** Summary of Findings for Other Outcomes	12
**Results S1:** Meta-analyses and narrative syntheses for other outcomes	14

Methods S1: Medline Database Search Strategy

Database: OVID Medline Epub Ahead of Print, In-Process & Other Non-Indexed Citations, Ovid MEDLINE(R) Daily and Ovid MEDLINE(R) 1946 to Present
Search Strategy:
----
1 bariatric surgery/ or gastric bypass/ or gastroplasty/ or jejunoileal bypass/ (27909)
2 (bariatric surg* or metabolic surger* or stomach stapling or jejuno-ileal bypass* or jejunoileal bypass* or gastroplasty* or gastric bypass* or gastroileal bypass* or gastrojejunostomy* or gastrogastostom* or gastro-gastostom* or restrictive surger* or gastric stapling or gastrointestinal surg* or gastrointestinal diversion* or sleeve gastrectom*).mp. [mp=title, book title, abstract, original title, name of substance word, subject heading word, floating sub-heading word, keyword heading word, organism supplementary concept word, protocol supplementary concept word, rare disease supplementary concept word, unique identifier, synonyms] (44863)
3 Biliopancreatic Diversion/ (1093)
4 (biliopancreatic diversion or bilio-pancreatic diversion or biliopancreatic bypass* or Bilio-pancreatic bypass*).mp. [mp=title, book title, abstract, original title, name of substance word, subject heading word, floating sub-heading word, keyword heading word, organism supplementary concept word, protocol supplementary concept word, rare disease supplementary concept word, unique identifier, synonyms] (1717)
5 (gastric band* or silicon band*).mp. (4136)
6 gastroenterostomy/ (3610)
7 (gastrectom* or gastroplast* or LAGB or stomach stapl* or lap band* or malabsorptive surg* or mason* procedure).mp. [mp=title, book title, abstract, original title, name of substance word, subject heading word, floating sub-heading word, keyword heading word, organism supplementary concept word, protocol supplementary concept word, rare disease supplementary concept word, unique identifier, synonyms] (56885)
8 (malabsorptive procedur* or "Roux-en-Y" or RYGB or duodenal switch* or stomach stapl*).mp. (4898)
9 Obesity/su [Surgery] (6815)
10 or/1-9 (90895)
11 mental disorders/ or anxiety disorders/ or agoraphobia/ or anxiety, separation/ or neurocirculatory asthenia/ or neurotic disorders/ or obsessive-compulsive disorder/ or hoarding disorder/ or panic disorder/ or phobic disorders/ or phobia, social/ or "bipolar and related disorders"/ or bipolar disorder/ or "disruptive, impulse control, and conduct disorders"/ or firesetting behavior/ or gambling/ or trichotillomania/ or dissociative disorders/ or dissociative identity disorder/ or elimination disorders/ or encopresis/ or enuresis/ or diurnal enuresis/ or nocturnal enuresis/ or "feeding and eating disorders"/ or anorexia nervosa/ or avoidant restrictive food intake disorder/ or binge-eating disorder/ or bulimia nervosa/ or diabulimia/ or "feeding and eating disorders of childhood"/ or food addiction/ or night eating syndrome/ or orthorexia nervosa/ or pica/ or relative energy deficiency in sport/ or female athlete triad syndrome/ or rumination syndrome/ or mood disorders/ or depressive disorder/ or depression, postpartum/ or depressive disorder, major/ or depressive disorder, treatment-resistant/ or dysthymic disorder/ or premenstrual dysphoric disorder/ or seasonal affective disorder/ or vascular depression/ or cyclothymic disorder/ or motor disorders/ or neurocognitive disorders/ or amnesia/ or alcoholic korsakoff syndrome/ or amnesia, anterograde/ or amnesia, retrograde/ or amnesia, transient global/ or cognition disorders/ or auditory perceptual disorders/ or huntington disease/ or cognitive dysfunction/ or chemotherapy-related cognitive impairment/ or postoperative cognitive complications/ or consciousness disorders/ or delirium/ or emergence delirium/ or dementia/ or aids dementia complex/ or alzheimer disease/ or aphasia, primary progressive/ or primary progressive nonfluent aphasia/ or creutzfeldt-jakob syndrome/ or dementia, vascular/ or dementia, multi-infarct/ or diffuse neurofibrillary tangles with calcification/ or frontotemporal lobar degeneration/ or frontotemporal dementia/ or "pick disease of the brain"/ or kluver-bucy syndrome/ or lewy body disease/ or dyslexia, acquired/ or alexia, pure/ or neurodevelopmental disorders/ or "attention deficit and disruptive behavior disorders"/ or attention deficit disorder with hyperactivity/ or conduct disorder/ or sluggish cognitive tempo/ or child behavior disorders/ or child development disorders, pervasive/ or autism spectrum disorder/ or asperger syndrome/ or autistic disorder/ or communication disorders/ or childhood-onset fluency disorder/ or social communication disorder/ or speech sound disorder/ or developmental disabilities/ or intellectual disability/ or learning disabilities/ or dyscalculia/ or dyslexia/ or specific learning disorder/ or motor skills disorders/ or mutism/ or reactive attachment disorder/ or schizophrenia, childhood/ or stereotypic movement disorder/ or tic disorders/ or tourette syndrome/ or paraphilic disorders/ or exhibitionism/ or fetishism, psychiatric/ or masochism/ or pedophilia/ or sadism/ or transvestism/ or voyeurism/ or personality disorders/ or antisocial personality disorder/ or borderline personality disorder/ or compulsive personality disorder/ or dependent personality disorder/ or histrionic personality disorder/ or hysteria/ or paranoid personality disorder/ or passive-aggressive personality disorder/ or schizoid personality disorder/ or schizotypal personality disorder/ or "schizophrenia spectrum and other psychotic disorders"/ or affective disorders, psychotic/ or capgras syndrome/ or delusional parasitosis/ or morgellons disease/ or paranoid disorders/ or psychotic disorders/ or psychoses, substance-induced/ or psychoses, alcoholic/ or schizophrenia/ or schizophrenia, catatonic/ or

schizophrenia, disorganized/ or schizophrenia, paranoid/ or schizophrenia, treatment-resistant/ or shared paranoid disorder/ or sexual dysfunctions, psychological/ or dyspareunia/ or erectile dysfunction/ or gender dysphoria/ or premature ejaculation/ or "sexual and gender disorders"/ or vaginismus/ or sleep wake disorders/ or dyssomnias/ or sleep deprivation/ or sleep disorders, circadian rhythm/ or jet lag syndrome/ or sleep disorders, intrinsic/ or "disorders of excessive somnolence"/ or idiopathic hypersomnia/ or kleine-levin syndrome/ or narcolepsy/ or cataplexy/ or restless legs syndrome/ or "sleep initiation and maintenance disorders"/ or parasomnias/ or nocturnal paroxysmal dystonia/ or rem sleep parasomnias/ or rem sleep behavior disorder/ or sleep paralysis/ or sleep arousal disorders/ or night terrors/ or somnambulism/ or sleep bruxism/ or sleep-wake transition disorders/ or somatoform disorders/ or body dysmorphic disorders/ or body integrity identity disorder/ or conversion disorder/ or globus sensation/ or factitious disorders/ or munchausen syndrome/ or munchausen syndrome by proxy/ or hypochondriasis/ or neurasthenia/ or substance-related disorders/ or alcohol-related disorders/ or alcohol amnestic disorder/ or alcohol withdrawal delirium/ or alcoholic intoxication/ or alcoholism/ or binge drinking/ or wernicke encephalopathy/ or amphetamine-related disorders/ or cocaine-related disorders/ or inhalant abuse/ or marijuana abuse/ or "marijuana use"/ or narcotic-related disorders/ or opioid-induced constipation/ or opioid-related disorders/ or heroin dependence/ or morphine dependence/ or opiate overdose/ or opium dependence/ or neonatal abstinence syndrome/ or phencyclidine abuse/ or substance abuse, intravenous/ or substance abuse, oral/ or substance withdrawal syndrome/ or "tobacco use disorder"/ or "trauma and stressor related disorders"/ or adjustment disorders/ or stress disorders, traumatic/ or battered child syndrome/ or combat disorders/ or psychological trauma/ or historical trauma/ or sexual trauma/ or stress disorders, post-traumatic/ or stress disorders, traumatic, acute/ (1389839)

12 (mental disorder* or mental disease* or anxi* or agoraphobi* or neurotic* or neuros* or obsessive-compulsive* or OCD or hoarding or phobi* or trichotillomania).mp. (729795)

13 (elimination disorder* or eating disorder* or anorexi* or binge or bulimi* or food addict* or night eating or pica or "loss of control" or disordered eat* or overvaluation).mp. (83942)

14 (bipolar or mania or manic or mood disorder* or depress* or dysthym*or premenstrual dysphoric disorder* or seasonal affective disorder* or vascular depressi* or cyclothymi*).mp. (692164)

15 (motor disorder* or neurocognitive disorder* or amnesia or korsakoff* or wernicke* or amnesia, or cognition disorder* or cognitive disorder* or auditory perceptual disorder* or huntington* or cognitive dysfunction* or consciousness disorder* or dementia or alzheimer* or frontotemporal dementia or lewy body or dyslexia).mp. (417870)

16 (neurodevelopmental disorder* or attention deficit or ADHD or conduct disorder or autism or asperger* or autistic or communication disorder* or developmental disabilit* or intellectual disabilit* or learning disabilit* or tic disorder* or tourette*).mp. (222746)

17 (personality disorder* or antisocial or borderline personality disorder* or BPD or borderline or hysteri* or histrionic or dissociat*).mp. (274037)

18 (schizo* or psychotic or psychosis or psychoses or catatoni* or paranoi* or delusion*).mp. (256544)

19 (sleep wake disorder* or sleep disorder* or circadian rhythm or hypersomni* or narcolepsy or cataplexy or restless legs syndrome* or parasomnia* or night terror*).mp. (134789)

20 (somatoform disorder* or somatic or somatize or body dysmorphi* or body integrity identity disorder* or conversion or hypochondria*).mp. (386366)

21 (substance misuse or substance abuse or substance dependen*).mp. (62377)

22 (alcoholism or alcohol withdrawal or alcoholic intoxication or binge drink* or alcohol or amphetamine or methamphetamine or methylphenidate or cocaine or marijuana abuse or marijuana or cannabis or narcotic or opioid or heroin or morphine or opiate or phencyclidine or tobacco or nicotine).mp. (827489)

23 (stress or trauma* or PTSD).mp. (1499175)

24 Suicide/ or Suicide, Completed/ or Suicide, Attempted/ or Self-Injurious Behaviour/ or Suicidal Ideation/ (66172)

25 (suicid* or self-injur* or self-harm).mp. (117014)

26 neuropsychological tests/ or bender-gestalt test/ or language tests/ or luria-nebraska neuropsychological battery/ or "memory and learning tests"/ or mental navigation tests/ or "mental status and dementia tests"/ or psychiatric status rating scales/ or stroop test/ or trail making test/ or wisconsin card sorting test/ (185300)

27 cognition/ or awareness/ or cognitive reserve/ or comprehension/ or consciousness/ or imagination/ or metacognition/ (177687)

28 Cognitive Aging/ (1026)

29 (neuropsych* or memory or processing speed or executive function or cogniti* or comprehen* or metacogniti*).mp. (1289496)

30 (brain adj3 (age or aging or health)).mp. (15139)

31 ((cognit* or memor* or brain) adj4 (improv* or function* or process* or test* or decline or impair* or performance)).mp. (406757)

32 or/11-31 (5601507)

33 obesity/ or obesity, abdominal/ or obesity, maternal/ or obesity, metabolically benign/ or obesity, morbid/ or pediatric obesity/ or prader-willi syndrome/ (248291)

34 (obesity or obese or prader-willi syndrome).mp. (416303)

35 Overweight/ (31020)
36 (overweight or (over adj weight)).mp. (88227)
37 Weight Loss/ (41938)
38 (weight adj2 loss).mp. (116949)
39 or/33-38 (504075)
40 10 and 32 and 39 (6315)
41 animals/ not humans/ (5009816)
42 40 not 41 (6187)
43 historical article/ or biography/ or blog/ or interview/ or personal narrative/ or autobiography/ or classical article/ or festschrift/ (408821)
44 Letter/ (1192436)
45 Comment/ (977915)
46 Editorial/ (619169)
47 (letter* or comment* or editorial or autobiography or blog*).mp. (2304748)
48 ((literature or scoping or narrative) adj3 review*).mp. (392348)
49 or/43-48 (3059126)
50 42 not 49 (5736)
51 exp Neoplasms/ (3732396)
52 (neoplas* or cancer* or tumo?r* or carcinoma* or malignan*).mp. [mp=title, book title, abstract, original title, name of substance word, subject heading word, floating sub-heading word, keyword heading word, organism supplementary concept word, protocol supplementary concept word, rare disease supplementary concept word, unique identifier, synonyms] (4677122)
53 51 or 52 (5055680)
54 50 not 53 (5359)

Table S1: Hierarchy of Outcome Selection Table

Outcome	Hierarchy
**All meta-analyses**	Largest sample size > longest follow-up
**Non-normative eating symptoms**	Total disordered eating symptom score > binge eating > loss of control eating/disinhibition > emotional eating
**Eating disorder**	Any eating disorder > binge eating disorder > loss of control over eating > objective binge eating episodes
**Any substance use**	Alcohol use > tobacco use > cannabis use
**Substance use symptoms**	Alcohol use disorder identification test > drug use disorders identification test
**Probable substance use disorder**	Any substance use disorder > alcohol use disorder > other
**Suicidal ideation or behaviour**	Any suicidal ideation or behaviour > any suicidal behaviour > lifetime suicide attempt > suicidal ideation past year > suicidal ideation current
**Memory**	General memory > memory subscales > verbal learning > visuospatial memory > immediate recall
**Executive function**	General executive function > switching attention > Stroop test > digit span > cognitive flexibility > decision making
**Attention**	General attention > working memory > processing speed > complex attention
**Psychiatric treatment**	Any psychotropic > any antidepressant > any anxiolytic > continued depression treatment (depression history) > new depression treatment (no depression history)
**Psychiatric hospitalization**	Any psychiatric diagnosis > alcohol use disorder diagnosis

Table S2: Evidence Profile for all Outcomes

Certainty assessment							Summary of findings				
							Study event rates (%)		Relative effect (95% CI)	Anticipated absolute effects	
Participants (studies) Follow-up	Risk of bias	Inconsistency	Indirectness	Imprecision	Publication bias	Overall certainty of evidence	With no intervention	With bariatric surgery		Risk with no intervention	Risk difference with bariatric surgery
**Depressive symptoms (follow-up: range 12 to 24 months)**											
93 (2 RCTs)	serious[a]	not serious	not serious	very serious[b]	none	⨁◯◯◯ Very low[a,b]	47	46	-	-	SMD **0.4 lower** (1.04 lower to 0.24 higher)
**Depressive symptoms (follow-up: range 3 to 58 months)**											
2003 (18 NRS)	very serious[c]	not serious	not serious	not serious	none	⨁⨁◯◯ Low[c]	960	1043	-	-	SMD **0.56 SD lower** (0.87 lower to 0.26 lower)
**Anxiety symptoms (follow-up: range 3 to 108 months)**											
1166 (11 NRS)	very serious[c]	not serious	not serious	not serious	none	⨁⨁◯◯ Low[c]	507	659	-	-	SMD **0.6 lower** (1 lower to 0.19 lower)
**Non-normative eating symptoms (follow-up: range 6 to 58 months)**											
6066 (15 NRS)	very serious[c]	not serious	not serious	not serious	none	⨁⨁◯◯ Low[c]	2893	3173	-	-	SMD **0.75 SD lower** (0.97 lower to 0.53 lower)

Certainty assessment							Summary of findings				
**Substance use disorder proportion ≤2 years of follow-up**											
151431 (5 NRS)	serious[d]	not serious	not serious	not serious	none	⊕⊕⊕◯ Moderate[d]	3061/84726 (3.6%)	1595/66705 (2.4%)	**RR 0.97** (0.64 to 1.46)	3061/84726 (3.6%)	**0 fewer per 100** (from 1 fewer to 2 more)
**Substance use disorder proportion >2 years of follow-up**											
193959 (8 NRS)	serious[d]	serious[e]	not serious	not serious	none	⊕⊕◯◯ Low[d,e]	4303/115584 (3.7%)	4049/78375 (5.2%)	**RR 2.13** (1.33 to 3.42)	4303/115584 (3.7%)	**4 more per 100** (from 1 more to 9 more)
**Suicide death proportion (follow-up: range 4 to 21 years)**											
167042 (5 NRS)	very serious[c]	not serious	not serious	serious[f]	none	⊕◯◯◯ Very low[c,f]	158/102994 (0.24%)	153/64048 (0.15%)	**RR 1.86** (1.07 to 3.21)	158/102994 (0.24%)	**1 more per 1,000*** (from 0 fewer to 3 more)
**Attention (follow-up: range 3 to 24 months)**											
271 (4 NRS)	very serious[c]	not serious	not serious	serious[g]	none	⊕◯◯◯ Very low[c,g]	123	148	-	-	SMD **0.72 higher** (0.17 lower to 1.61 higher)
**Executive function (follow-up: range 3 to 24 months)**											
515 (6 NRS)	very serious[c]	not serious	not serious	serious[h]	none	⊕◯◯◯ Very low[c,h]	244	271	-	-	SMD **0.14 higher** (0.04 lower to 0.32 higher)

Certainty assessment							Summary of findings				
**Memory (follow-up: range 3 to 24 months)**											
298 (4 NRS)	very serious[c]	not serious	not serious	serious[g]	none	⊕◯◯◯ Very low[c,g]	124	174	-	-	SMD **0.17 higher** (0.29 lower to 0.62 higher)
**Depressive disorder proportion (follow-up: range 2 to 5 years)**											
133 (2 RCTs)	serious[a]	not serious	not serious	very serious[i]	none	⊕◯◯◯ Very low[a,i]	8/63 (12.7%)	9/70 (12.9%)	**RR 0.99** (0.41 to 2.41)	8/63 (12.7%)	**0 fewer per 100** (from 7 fewer to 18 more)
**Depressive disorder proportion (follow-up: range 8 to 120 months)**											
987503 (13 NRS)	very serious[c]	not serious	not serious	not serious	none	⊕⊕◯◯ Low[c]	76524/830604 (9.2%)	19167/156899 (12.2%)	**RR 1.07** (0.94 to 1.21)	76524/830604 (9.2%)	**1 more per 100** (from 1 fewer to 2 more)
**Anxiety disorder proportion (follow-up: range 1 to 9 years)**											
115060 (5 NRS)	very serious[c]	not serious	not serious	not serious	none	⊕⊕◯◯ Low[c]	8025/57549 (13.9%)	9304/57511 (16.2%)	**RR 1.16** (1.13 to 1.19)	8025/57549 (13.9%)	**2 more per 100** (from 2 more to 3 more)
**Eating disorder proportion (follow-up: range 8 to 108 months)**											
115084 (10 NRS)	very serious[c]	serious[j]	not serious	not serious	publication bias strongly suspected[k]	⊕◯◯◯ Very low[c,j,k]	491/57584 (0.9%)	2574/57500 (4.5%)	**RR 5.32** (4.84 to 5.86)	491/57584 (0.9%)	**4 more per 100** (from 3 more to 4 more)

Certainty assessment							Summary of findings				
**Any substance use proportion (follow-up: range 12 to 24 months)**											
90 (2 RCTs)	serious[a]	not serious	not serious	very serious[i]	none	⊕◯◯◯ Very low[a,i]	11/44 (25.0%)	9/46 (19.6%)	**RR 0.80** (0.33 to 1.98)	11/44 (25.0%)	**5 fewer per 100** (from 17 fewer to 25 more)
**Any substance use proportion (follow-up: range 2 to 8 years)**											
79780 (4 NRS)	very serious[c]	serious[e]	not serious	not serious	none	⊕◯◯◯ Very low[c,e]	10431/46260 (22.5%)	5298/33520 (15.8%)	**RR 1.22** (1.04 to 1.42)	10431/46260 (22.5%)	**5 more per 100** (from 1 more to 9 more)
**Substance use symptoms (follow-up: mean 8 years)**											
26840 (1 NRS)	serious[l]	not serious	serious[m]	not serious	none	⊕⊕◯◯ Low[l,m]	24232	2608	-	24232	MD **0.3 AUDIT-C score higher** (0.18 higher to 0.42 higher)
**Suicidal ideation and self-harm behaviour proportion ≤2 years of follow-up**											
113545 (2 NRS)	very serious[c]	not serious	not serious	not serious	none	⊕⊕◯◯ Low[c]	577/56731 (1.0%)	262/56814 (0.5%)	**RR 0.45** (0.39 to 0.52)	577/56731 (1.0%)	**1 fewer per 100** (from 1 fewer to 0 fewer)
**Suicidal ideation and self-harm behaviour proportion >2 years of follow-up**											
84982 (4 NRS)	serious[d]	not serious	not serious	not serious	none	⊕⊕⊕◯ Moderate[d]	1973/57784 (3.4%)	708/27198 (2.6%)	**RR 1.02** (0.93 to 1.11)	1973/57784 (3.4%)	**0 fewer per 100** (from 0 fewer to 0 fewer)

Certainty assessment							Summary of findings				
**Dementia proportion (follow-up: range 1 to 10 years)**											
192748 (5 NRS)	very serious[c]	not serious	not serious	not serious	none	⊕⊕◯◯ Low[c]	744/104053 (0.7%)	678/88695 (0.8%)	**RR 0.89** (0.39 to 2.01)	744/104053 (0.7%)	**0 fewer per 100** (from 0 fewer to 1 more)
**Psychiatric treatment use proportion (follow-up: range 2 to 7 years)**											
71695 (9 NRS)	serious[d]	not serious	not serious	not serious	none	⊕⊕⊕◯ Moderate[d]	19355/47736 (40.5%)	6937/23959 (29.0%)	**RR 1.22** (1.08 to 1.38)	19355/47736 (40.5%)	**9 more per 100** (from 3 more to 15 more)
**Psychiatric hospitalization proportion (follow-up: range 2 to 9 years)**											
23860 (5 NRS)	very serious[c]	not serious	not serious	not serious	none	⊕⊕◯◯ Low[c]	472/10265 (4.6%)	501/13595 (3.7%)	**RR 1.17** (0.98 to 1.40)	472/10265 (4.6%)	**1 more per 100** (from 0 fewer to 2 more)
**Any psychiatric disorder proportion (follow-up: range 1 to 9 years)**											
113686 (3 NRS)	very serious[c]	not serious	not serious	not serious	none	⊕⊕◯◯ Low[c]	26357/56866 (46.3%)	39809/56820 (70.1%)	**RR 1.68** (1.34 to 2.10)	26357/56866 (46.3%)	**32 more per 100** (from 16 more to 51 more)

CI: confidence interval; **MD:** mean difference; **NRS:** non-randomized studies; **RCTs:** randomized controlled studies; **RR:** risk ratio; **SMD:** standardised mean difference.
*****denominator is out of 1000 people

Explanations

a. Around half of the studies in this meta-analysis are at high risk of bias.
b. The 95%CI crosses a large reduction (SMD<-0.8) and small increase (SMD>0.2). Inconsistency is likely driving some of this imprecision, though did not downgrade separately for inconsistency.
c. All studies are at either serious or critical risk of bias overall.
d. Most studies are at either serious or critical risk of bias overall.
e. Considerable heterogeneity (I^2>90%, many studies have substantially different effects from the point estimate) that is likely leading to imprecision (the 95%CI crosses the threshold of a difference in 2/100 people).

f. The 95%CI crosses the threshold of a difference in 1/1000 people. Inconsistency is likely driving some of the imprecision, though did not downgrade separately for inconsistency.
g. The 95%CI crosses the threshold for appreciable benefit (SMD>0.2). Inconsistency is likely driving some of this imprecision, though did not downgrade separately for inconsistency.
h. The 95%CI crosses the threshold for appreciable benefit (SMD>0.2).
i. The 95%CI crosses a reduction (2/100 fewer people) and an increase (2/100 more people).
j. Considerable heterogeneity (I^2=98%, many studies have substantially different effects from the point estimate).
k. Funnel plot suggests publication bias.
l. All studies at serious risk of bias overall.
m. Meta-analysis is derived from a single study, with multiple different study populations.

Table S3. Summary of Findings for Other Outcomes

Outcomes	**Anticipated absolute effects*** (95% CI)		Relative effect (95% CI)	№ of participants (studies)	Certainty of the evidence (GRADE)	Comments
	**Risk with no intervention**	**Risk with bariatric surgery**				
Depressive disorder proportion follow-up: range 2 to 5 years	13 per 100	**13 per 100** (5 to 31)	**RR 0.99** (0.41 to 2.41)	133 (2 RCTs)	⊕◯◯◯ Very low[a,e]	MBS may have little to no effect on depressive disorders, but the evidence is very uncertain.
Depressive disorder proportion follow-up: range 8 to 120 months	9 per 100	**10 per 100** (9 to 11)	**RR 1.07** (0.94 to 1.21)	987503 (13 NRS)	⊕⊕◯◯ Low[b]	MBS may not increase depressive disorders.
Anxiety disorder proportion follow-up: range 1 to 9 years	14 per 100	**16 per 100** (16 to 17)	**RR 1.16** (1.13 to 1.19)	115060 (5 NRS)	⊕⊕◯◯ Low[b]	MBS may slightly increase anxiety disorders.
Eating disorder proportion follow-up: range 8 to 108 months	1 per 100	**5 per 100** (4 to 5)	**RR 5.32** (4.84 to 5.86)	115084 (10 NRS)	⊕◯◯◯ Very low[b,f,g]	MBS may slightly increase eating disorders, but the evidence is very uncertain.
Any substance use proportion follow-up: range 12 to 24 months	25 per 100	**20 per 100** (8 to 50)	**RR 0.80** (0.33 to 1.98)	90 (2 RCTs)	⊕◯◯◯ Very low[a,e]	MBS may slightly reduce substance use, but the evidence is very uncertain.
Any substance use proportion follow-up: range 2 to 8 years	23 per 100	**28 per 100** (23 to 32)	**RR 1.22** (1.04 to 1.42)	79780 (4 NRS)	⊕◯◯◯ Very low[b,d]	MBS may slightly increase substance use, but the evidence is very uncertain.
Substance use symptoms follow-up: mean 8 years	The mean substance use symptoms was **1.39** AUDIT-C score	MD **0.3 AUDIT-C score higher** (0.18 higher to 0.42 higher)	-	26840 (1 NRS)	⊕⊕◯◯ Low[h,i]	MBS may not increase substance use symptoms.
Suicidal ideation and self-harm behaviour proportion ≤2 years of follow-up	1 per 100	**0 per 100** (0 to 1)	**RR 0.45** (0.39 to 0.52)	113545 (2 NRS)	⊕⊕◯◯ Low[b]	MBS may not reduce suicidal ideation and self-harm within 2 years of follow-up.
Suicidal ideation and self-harm behaviour proportion >2 years of follow-up	3 per 100	**3 per 100** (3 to 4)	**RR 1.02** (0.93 to 1.11)	84982 (4 NRS)	⊕⊕⊕◯ Moderate[c]	MBS probably does not increase suicidal ideation and self-harm behaviour after 2 years of follow-up.
Dementia proportion follow-up: range 1 to 10 years	1 per 100	**1 per 100** (0 to 1)	**RR 0.89** (0.39 to 2.01)	192748 (5 NRS)	⊕⊕◯◯ Low[b]	MBS may not reduce dementia.
Psychiatric treatment use proportion follow-up: range 2 to 7 years	41 per 100	**49 per 100** (44 to 56)	**RR 1.22** (1.08 to 1.38)	71695 (9 NRS)	⊕⊕⊕◯ Moderate[c]	MBS probably increases psychiatric treatment use slightly.
Psychiatric hospitalization proportion follow-up: range 2 to 9 years	5 per 100	**5 per 100** (5 to 6)	**RR 1.17** (0.98 to 1.40)	23860 (5 NRS)	⊕⊕◯◯ Low[b]	MBS may not increase psychiatric hospitalizations.

Outcomes	Anticipated absolute effects* (95% CI)		Relative effect (95% CI)	№ of participants (studies)	Certainty of the evidence (GRADE)	Comments
	Risk with no intervention	Risk with bariatric surgery				
Any psychiatric disorder proportion follow-up: range 1 to 9 years	46 per 100	**78 per 100** (62 to 97)	**RR 1.68** (1.34 to 2.10)	113686 (3 NRS)	⊕⊕◯◯ Low[b]	MBS may result in a large increase in psychiatric disorder diagnoses.

***The risk in the intervention group** (and its 95% confidence interval) is based on the assumed risk in the comparison group and the **relative effect** of the intervention (and its 95% CI).

CI: confidence interval; **MD:** mean difference; **RR:** risk ratio; **SMD:** standardised mean difference

GRADE Working Group grades of evidence
High certainty: we are very confident that the true effect lies close to that of the estimate of the effect.
Moderate certainty: we are moderately confident in the effect estimate: the true effect is likely to be close to the estimate of the effect, but there is a possibility that it is substantially different.
Low certainty: our confidence in the effect estimate is limited: the true effect may be substantially different from the estimate of the effect.
Very low certainty: we have very little confidence in the effect estimate: the true effect is likely to be substantially different from the estimate of effect.

Explanations

a. Around half of the studies in this meta-analysis are at high risk of bias.
b. All studies are at either serious or critical risk of bias overall.
c. Most studies are at either serious or critical risk of bias overall.
d. Considerable heterogeneity (I2=90%, many studies have substantially different effect from the point estimate) that is likely leading to imprecision (the 95%CI crosses the threshold of a difference in 2/100 people).
e. The 95%CI crosses a reduction (2/100 fewer people) and an increase (2/100 more people).
f. Considerable heterogeneity (I2>85%, many studies have substantially different effect from the point estimate)
g. Funnel plot suggests publication bias
h. All studies at serious risk of bias overall
i. Meta-analysis is derived from a single study, with multiple different study populations

Results S1: Meta-analyses and narrative syntheses for other outcomes

Depressive disorder
From meta-analyses, MBS had little to no effect on depressive disorders in 2 RCTs of 133 people (RR=0.99, 95%CI 0.41-2.41; 0/100 fewer people, from 7 fewer to 18 more) or 5 NRS of 987,503 people (RR=1.07, 95%CI 0.94-1.21; 1/100 more people, 95%CI from 1 fewer to 2 more). No sensitivity analyses were possible. Vote counting in 2 NRS[1,2] also suggested there was no evidence of benefit (p=0.75). Certainty in these outcomes is very low and low, respectively.

Anxiety disorder
From a meta-analysis of 58,479 people from 5 NRS, MBS may slightly increase anxiety disorders 1-9 years after surgery (RR=1.16, 95%CI 1.13-1.19; 2/100 more people, 95%CI from 2 to 3 more), however, certainty is low. Consistency was found in a sensitivity analysis removing studies with passive comparators. An NRS ineligible for synthesis aligned with the direction of effect in the meta-analysis,[2] as did a RCT.[3]

Eating disorder
From a meta-analysis of 115,084 people from 10 NRS, MBS may slightly increase eating disorders after 8-108 months (RR=5.32, 95%CI 4.84-5.86; 4/100 more people, from 3 to 4 more; fixed effects model), but the evidence is very uncertain. Results were similar in a sensitivity analysis removing studies at critical risk of bias but showed a reversed effect when studies with passive comparators were removed (RR=0.32, 95%CI 0.11-0.91). One NRS[1] and one RCT[4] were ineligible for synthesis and neither were consistent with the direction of effect in the meta-analysis.

Any Substance Use
From a meta-analysis of 95 people from 2 RCTs, MBS may slightly reduce nicotine use over 12-24 months (RR=0.80, 95%CI 0.33-1.98; 5/100 fewer people, 95%CI from 17 fewer to 25 more), but the evidence is very uncertain. No sensitivity analyses were possible. In a meta-analysis of 79,780 people from 4 NRS, MBS may slightly increase any substance over 2-8 years (RR=1.22, 95%CI 1.04-1.42; 5/100 more people, 95%CI from 1 to 9 more), but the evidence is very uncertain. Results were similar in a sensitivity analysis removing studies with passive comparators.

Substance Use Symptoms
From a meta-analysis of 26,840 people, combining 4 study populations from 1 NRS, MBS may not increase AUDIT-C scores after 8 years (MD=0.30, 95%CI 0.18-0.42), however, certainty is low. No sensitivity analyses were possible. Little to no effect on AUDIT scores were reported in a NRS (SMD=0.14)[5] and a RCT (MD=-1.04, 95%CI -2.42, 0.34)[4] that were ineligible for synthesis.

Suicidal Ideation/Non-fatal Self-harm Behavior
MBS had no effect on suicidal ideation/non-fatal self-harm behavior in an overall meta-analysis (RR=0.79, 95%CI 0.35-1.79; 0/100 fewer people, 95%CI from 1 fewer to 2 more), however, heterogeneity was high (I^2=97%), and there was a significant subgroup effect (test for subgroup difference p=0.003) based on duration of follow-up. From a subgroup meta-analysis of 113,545 people from 2 NRS, MBS may not reduce suicidal ideation/non-fatal self-harm behavior within 2 years (RR=0.45, 95%CI 0.39-0.52; 1/100 fewer people, 95%CI from 1 to 0 fewer; fixed effects model), however, certainty is low. From a subgroup meta-analysis of 84,982 people from 4 NRS, MBS probably does not increase suicidal ideation/non-fatal self-harm behavior after 2 years (RR=1.02, 95%CI 0.93-1.11; 0/100 fewer people, 95%CI from 0 fewer to 0 more; fixed effects model; moderate certainty). No sensitivity analyses were possible. A RCT was consistent with the direction of effect in the latter meta-analysis,[4] and combining p-values in two NRS also suggested there was no effect of surgery on suicidal ideation/non-fatal self-harm behavior (p=0.25)[2,6].

Dementia
From a meta-analysis of 192,748 people from 5 NRS, MBS may not reduce dementia over 1-10 years (RR=0.89, 95%CI 0.39-2.01; 0/100 fewer people, from 0 fewer to 1 more), however, certainty is low and results were consistent in a sensitivity analysis removing a study at critical risk of bias (RR=1.00, 95%CI 0.37-2.66). A RCT was consistent with the meta-analysis.[3] Definitions of dementia varied by study and included any dementia (3 studies), hospitalization for dementia (1 study), any memory impairment (1 study) and organic mental disorder including unspecified dementia or delirium (1 study).

Psychiatric Treatment Use

From a meta-analysis of 71,695 people from 9 NRS, MBS probably increases psychiatric treatment use slightly over 2-7 years (RR=1.22, 95%CI 1.08-1.38; 9/100 more, 95%CI from 3 to 15 more; moderate certainty). Sensitivity analyses showed consistent results. Types of treatment included in this composite included antidepressant use (5 studies), antidepressants or psychotherapy use (2 studies), any psychotropic use (1 study), and substance use disorder treatment use (1 study).

Psychiatric Hospitalization

From a meta-analysis of 23,860 people from 5 NRS, MBS may not increase hospitalizations for psychiatric reasons over 2-9 years (RR=1.17, 95%CI 0.98-1.40; 1/100 more, 95%CI from 0 fewer to 2 more), however, certainty is low. Sensitivity analyses showed consistent results. Reasons for hospitalization included any psychiatric disorder (3 studies), alcohol-related complications (1 study), and self-harm (1 study). A NRS that was ineligible for synthesis did not align with the meta-analysis, finding increased hospitalizations after MBS.[2]

Any Psychiatric Disorder

From a meta-analysis of 113,686 people from 3 NRS, MBS may result in a large increase in psychiatric disorder diagnoses after 1-9 years (RR=1.68, 95%CI 1.34-2.10; 32/100 more, 95%CI from 16 to 51 more), however, certainty is low. While a sensitivity analysis that removed a study with a passive comparator did not change the direction of effect, it reduced its magnitude to a small increase in diagnoses (RR=1.37, 95%CI 1.07-1.77; 4/100 more, 95%CI from 1 to 9 more).

References

1. Mirijello A, D'Angelo C, Iaconelli A, et al. Social phobia and quality of life in morbidly obese patients before and after bariatric surgery. *Journal of Affective Disorders*. 2015;179:95-100. doi:10.1016/j.jad.2015.03.030

2. Oh JS, Ham P, Drinane J, Lane B, Lee S. PREVALENCE OF PSYCHIATRIC DISORDERS IN HOSPITALIZED PATIENTS AFTER BARIATRIC SURGERY USING A NATIONAL DATABASE. In: ; 2016:119-120.

3. Cohen RV, Pereira TV, Aboud CM, et al. Gastric bypass versus best medical treatment for diabetic kidney disease: 5 years follow up of a single-centre open label randomised controlled trial. *eClinicalMedicine*. 2022;53:101725. doi:10.1016/j.eclinm.2022.101725

4. Järvholm K, Janson A, Peltonen M, et al. Metabolic and bariatric surgery versus intensive non-surgical treatment for adolescents with severe obesity (AMOS2): a multicentre, randomised, controlled trial in Sweden. *The Lancet Child & Adolescent Health*. 2023;7(4):249-260. doi:10.1016/S2352-4642(22)00373-X

5. Vangoitsenhoven R, Frederiks P, Gijbels B, et al. Long-term effects of gastric bypass surgery on psychosocial well-being and eating behavior: not all that glitters is gold. *Acta Clinica Belgica*. 2016;71(6):395-402. doi:10.1080/17843286.2016.1174393

6. Kovacs Z, Valentin JB, Nielsen RE. Risk of psychiatric disorders, self-harm behaviour and service use associated with bariatric surgery. *Acta Psychiatr Scand*. 2017;135(2):149-158. doi:10.1111/acps.12669
